# Optimization of fuzzy logic controller in the converter of a standalone solar power system using the firefly algorithm

**DOI:** 10.1038/s41598-026-41508-0

**Published:** 2026-02-23

**Authors:** Pegah Nouri, Mehrdad Ahmadi Kamarposhti, Tohid Nouri, Mehdi Radmehr

**Affiliations:** 1 Department of Electrical Engineering, Sar.C., Islamic Azad University, Sari, Iran; 2 Department of Electrical Engineering, Jo.C., Islamic Azad University, Jouybar, Iran

**Keywords:** Standalone solar system, Inverter, Fuzzy logic controller (FLC), Firefly Algorithm (FA), Mean Squared Error (MSE), Total Harmonic Distortion (THD), Energy science and technology, Engineering, Mathematics and computing

## Abstract

In single photovoltaic (PV) systems, inverters are highly important for transforming DC voltage into AC voltage with predetermined amplitude and frequency control. A good inverter must provide a stable output voltage and low harmonic distortion during potential load variation, and it must recover its stability in an efficient manner while maintaining the quality of power during disturbances. Fixed controllers usually do not provide satisfactory performance because of the inherent nonlinear characteristics of the systems, and this drives the adoption of intelligent control techniques. One area of interest is fuzzy logic controllers (FLCs), which can provide a good control measure of nonlinear dynamics and do not require use of a specific mathematical model. This study introduces the Firefly Algorithm to optimize fuzzy controller membership functions for improved voltage regulation, reduced MSE, and lower THD. The input and output membership functions are optimized in order to minimize the mean square error (MSE) of the output voltage. The proposed controller is tested for different load conditions including resistive loads, inductive loads, and non-linear loads; the performance is compared to a traditional fuzzy logic controller (FLC) as well as FLCs optimized using a Genetic Algorithm (GA) and Particle Swarm Optimization (PSO). Results from the simulation indicate the performance achieved from Firefly Algorithm (FA) is acceptable when compared with voltage regulation, harmonic distortion (THD), and dynamic responses for stand-alone photovoltaic systems. Simulation results indicate that the FA-optimized fuzzy logic controller produces a minimum total harmonic distortion of 2.89% and a mean square error of 0.0071, thus showing its superiority over the traditional PI and fuzzy logic controllers for various load conditions.

## Introduction

Nowadays, solar energy systems are renewable energy technology that leverages safe, renewable, and sustainable energy sources. A typical solar system uses solar panel, DC/DC converter, MPPT circuits, batteries, inverters, and more. The inverter converts DC voltage to AC voltage with controlled amplitude and frequency to satisfy the desired output. An important feature of an effective inverter is that it is capable of maintaining the amplitude and frequencies that are desired in all operating conditions at low output harmonic distortion, and it needs to restore the voltage to stability quickly and without experiencing power quality issues at the presence of disruptions. Inverter control methods may be considered either conventional or intelligent, the traditional methods not met performance requirements because of the nonlinearity of the system, which lead to the demand for intelligent control methods.

Intelligent control methods based on fuzzy logic are becoming more appealing than other alternatives, primarily because fuzzy logic tends to require less complex mathematical models. Fuzzy logic could be considered a reasonable approach to deal with nonlinear problems. In a fuzzy logic framework, the variables/membership functions are typically developed by a system expert and/or through experimentation.

Even though they are relatively easy to use, fuzzy controllers could lead to poor performance for the inverter, or worse, an unstable system, if the designer does not have the appropriate membership functions. In this case, the designer needs to have a good understanding of the system’s operational conditions to accomplish a good design, or metaheuristic algorithms could help find the best membership functions. After the designer has designed the membership functions, intelligent algorithms can fine-tune the membership functions. The selected algorithm should enable high accuracy and convergence speed in the context of developing an appropriate membership function^1^.

The extension of large penetration of renewables has raised other issues such as harmonic distortion in the output, poor efficiency of power converters, lack of stability for output power and reliability from the perspective of a power electronic converter. Researchers have proposed the application of different control methods to reduce its effect on power converters’ operation and performance. PI (proportional-integral) controllers were commonly adopted as the convertor control algorithm for instance. However, the performance of the PI controller is typically limited to small load disturbances, it is based on an accurate mathematical model of the real system, and the controller parameters must be tuned for acceptable performance.

Fuzzy logic controllers (FLCs) are increasingly being used in converter control designs because of their important advantage, they do not require an exact mathematical model of the system. A FLC’s performance is based on the rule base, the number of rules, and the membership function design, which is typically established through trial-and-error; a very time-consuming process. Instead, optimization methods can be used to aid in the design of fuzzy controllers. A newly available and efficient optimization methodology, which can be utilized, is metaheuristic and evolutionary optimization algorithms, particularly beneficial for the design of fuzzy logic controllers, as it helps to locate global optimum solutions. In addition to their global optimum solutions, the algorithms are stochastic and are helpful in avoiding the algorithm from being trapped in local optima, thus making them beneficial for tuning the membership functions to improve overall controller performance^2,3^.

Designing input and output membership functions for inverter control systems is fundamental. A poorly designed membership function may lead to an output voltage that is objectionable with respect to amplitude, frequency, or harmonic content. In this paper, the optimal design of these membership functions is solved with the Firefly Algorithm (FA) in order to dramatically reduce the consequences of manual design or trial-and-error design, as well as improve performance of the inverter system. This study uses and will implement the Firefly Algorithm (FA) to optimally design the fuzzy controller for an inverter. Among other significant characteristics of the FA is its convergence speed and accuracy. The purpose is to design fuzzy membership functions to have a fuzzy controller that operates properly and minimizes the objective function. This paper presents a new method for optimizing a fuzzy controller for a three-phase standalone inverter utilizing the Firefly Algorithm (FA). The principal contributions are as follows:

- Exploiting the Firefly Algorithm to calibrate the input-output membership functions for the fuzzy controller to minimize mean squared error (MSE) of voltage and keep total harmonic distortion (THD) low.
- Evaluating the serviceability of the controller with variables on the load (resistive, inductive and non-linear load) contrasting against conventional PI controllers and GA and Particle Swarm Optimized Fuzzy controller.
- Establishing a method that is fast and efficient in tracking the reference voltage and maintaining stability with abrupt dips in load without compromising power quality.
- Emphasizing a method to lessen the need for mathematically accurate system models, and outline a methodology that is applicable and appropriate towards fuzzy controller design for a stand-alone power system.

The main originality of this work lies in the systematic application of the Firefly Algorithm (FA) for the simultaneous optimization of both input and output membership functions of a fuzzy logic controller used in a three-phase standalone photovoltaic inverter. Unlike most existing studies that either rely on manual tuning, trial-and-error approaches, or optimize only partial controller parameters, the proposed method provides a fully automated and unified optimization framework. In addition, the proposed controller is validated under diverse operating conditions, including resistive, inductive, and nonlinear loads, which is rarely addressed comprehensively in previous works. The comparative evaluation with GA- and PSO-based fuzzy controllers further demonstrates the superiority of the FA in terms of mean squared error (MSE), total harmonic distortion (THD), and dynamic voltage response.

This paper is divided into six sections. Section  2 includes a review of previous research. Section  3 explains the basics of fuzzy logic concepts. Section  4 presents the Firefly Algorithm and how it was used in the design of the fuzzy controller. Section  5 presents the simulation results and their analysis. Results and discussion is presented in Sect.  6. Finally, Sect.  7 contains key findings and remarks to conclude the document.

## Literature review

Many studies on inverter control methods have been reported, and in this section, a review of some of the more recent and relevant references is given.

Proportional-Integral (PI) controllers have been regarded as traditional and common approaches to inverter control. In^2^, a conventional PI controller was introduced for inverter control, but the configuration of this controller has been based on the differential equations of the plant in order to achieve acceptable performance. Likewise, a PI controller was applied to a three-phase inverter, but there was no indication of the tuning method for the controller parameters in^3^. More recently, the focus has been shifted towards using metaheuristic and intelligent algorithms to optimize PI controller parameters to improve performance^4,5^.

Another traditional controller widely used for inverter control is the PID controller. Compared with the PI controller, PID control generally provides better performance, but it also faces the same design problems. Besides, like for PI controllers, some intelligent algorithms have also been employed for the optimization of the parameters of PID. In^6^, PSO was adopted, while in^7^, GA was used. Traditional controllers are usually limited to small load changes since they are based on a precise mathematical model of a real system at a specific load condition. Although their design is classified, traditional controllers can lose performance under severe or sudden load changes and can even lead to instability^8^.

To overcome these challenges, inverter systems have been utilizing different AI-centric controllers, such as ANN, fuzzy logic, and ANFIS^9^. In recent years, fuzzy controllers have been applied in controlling inverter applications; they are simple to use and very flexible in complicated systems, even when they do not use complicated mathematical models^10^. The fuzzy controllers are well-structured but need precise specification of the membership functions and the rule bases. Each of the rule base, number of rules, and shape of the membership functions can influence the performances of the fuzzy-based controller. The number of rules being too many (overly complex) or too sporadic (not enough rules) or the membership functions being poorly shaped, or rules defined poorly can lead to performance that is not suitable for application. Often each of these factors are determined by trial and error which can be inefficient and laborious. To overcome this limitation, several methods have been proposed in the literature, such as neuro-fuzzy models^11^, and intelligent optimization algorithms^12^. Neuro-fuzzy models need a huge quantity of training data, that is impossible to get for some systems^13^. Due to this fact, the utilized algorithm in the design process of the membership functions should be accurate and has fast convergence rate, in order to successfully avoid the design difficulties of fuzzy controllers.

In recent years, fuzzy control has made great progress, especially in the domain of photovoltaic systems. For instance in^14^, a hybrid system was developed with a combination of fuzzy logic and the incremental conductance algorithm to perform MPPT and to exhibit superior performance compared to the traditional approaches by utilizing new input signals such as the sum of the conductances and the variations in the input signal, namely, CSI&SInC. Besides, high levels of precision and efficiency were exhibited regardless of the change in ambient conditions. This is an important step toward enhancing PV system operation, especially when their operation environments are liable to exhibit changing conditions. For designing an inverter with MPPT capability, FLC with SVPWM was used in^15^. Simulations and experimental setups yielded results that ensured the proposed combined system enhances MPPT tracking accuracy and reduces THD of the inverter output, hence indicating that FLC design plays a vital role in PV systems where the operating conditions change significantly in very short periods.

According to research in^16^ advanced control methods can be developed by combining Type 2 Fuzzy Logic with GA optimisation techniques and multi-layered perceptron (MLP) neural networks (NN’s) in order to obtain MPPT tracking with respect to irradiation and temperature variations. The study indicated that both the accuracy and stability of PV systems were improved by combining these techniques with others based on ANFIS. This methodology is particularly applicable for PV systems operating under varied environmental conditions. In addition in^17^ developed a PV system where a bidirectional DC-DC Converter was integrated with ANFIS. The hybrid configuration provides not only optimal power tracking and management of battery charging and battery voltage but also adaptive responses to changes in energy demand and in environmental conditions. Regarding the quality of power and the speeds of harmonics contained within a PV inverter system^18^, presented a Fuzzy Control Scheme based upon rules, which was used to optimise the switching of IGBTs, thereby substantially reducing THD in output voltage and improving the quality of power generated from the PV system. There has been a growing trend of developing hybrid control techniques in recent years.

There was a combination of fuzzy control and sliding mode control with an adaptive exponential reaching law presented in^19^. This allows for high robustness in terms of uncertainty and variation in power flow. The combination of fuzzy control with sliding mode control provides fast dynamic response, as well as excellent stability in the system. A hybrid MPPT strategy was developed in^20^, combining Incremental Conductance (IncCond) control with a fuzzy logic controller (FLC) to obtain better accuracy in the tracking of maximum power points while varying irradiance and load conditions, therefore allowing for the optimisation of MPPT. Ref^21^. demonstrated how to design a fuzzy controller for an inverter. Through simulation results and results obtained from dSPACE hardware, the designed controller was able to eliminate fluctuations in the raw output voltage, and produce a stable output and low Total Harmonic Distortion (THD). The paper in^22^ designed a second-order fuzzy controller for a Unified Power Quality Conditioner (UPQC) inverter associated with photovoltaic (PV) systems. This design has shown to improve the quality of power, especially with the presence of nonlinear loads. This is an excellent example of how fuzzy logic can be used to manage complex power quality issues. A dynamic adjustment with fuzzy logic in combination with MPPT of the solar panel’s tilt angle was introduced in^23^, an innovative approach that increases energy generation by about 20% compared to a fixed-angle system and especially in unbalanced irradiation conditions, therefore proving adaptability and effectiveness in fuzzy logic for optimization of photovoltaic output. In^24^, a self-adaptive fuzzy-PID controller optimized using an improved fitness-guided optimization algorithm (IFGO) was proposed for active power filter applications, showing significant improvement in harmonic compensation. Similarly, type-2 fuzzy logic combined with discrete wavelet transform techniques has been employed for harmonic mitigation in distribution systems^25^. Moreover, advanced fuzzy-based optimization methods have been applied to nonlinear power systems, demonstrating superior dynamic response and robustness compared to conventional controllers^26^. In addition, hybrid fuzzy and evolutionary control techniques have been reported to enhance system stability and tracking accuracy in complex nonlinear environments^27^. These recent works confirm the effectiveness of fuzzy-based intelligent controllers and motivate the adoption of the Firefly Algorithm in the present study for optimizing the membership functions of the fuzzy logic controller.

Table 1 shows a summary of some of the recent studies on inverter control using traditional and intelligent controllers. As can be seen in Table 1, most of the previous studies have focused on optimizing fuzzy controllers for particular or certain types of load conditions. In this study, however, the Firefly Algorithm is used to optimize both input and output membership functions of a fuzzy logic controller for a three-phase inverter, improving mean squared error (MSE), total harmonic distortion (THD), and response time for variable types of resistive, inductive, and nonlinear loads. This further proves the practical originality and superiority of this study compared to previous ones.

**Table 1:** Summary of recent studies on inverter control using traditional and intelligent controllers.

Study	Controller / Algorithm	Load Types	Performance Metrics	Key Notes / Limitation
[2]	PI	–	Voltage Regulation	Based on plant equations
[3]	PI	–	Voltage Regulation	No tuning method
[4–5]	PI + Metaheuristic	–	Voltage Regulation	Improves PI performance
[6]	PID + PSO	–	Voltage Regulation	Limited to small load changes
[7]	PID + GA	–	Voltage Regulation	Limited to small load changes
[8]	Traditional	–	Stability	Can lose performance under sudden load changes
[9–10]	ANN / FLC / ANFIS	–	–	AI controllers overcome traditional limitations
[11–13]	Neuro-Fuzzy	–	–	Needs large training data
[14–15]	FLC + MPPT / SVPWM	PV variable	MPPT, THD	Improves tracking accuracy
[16–20]	Advanced Fuzzy / Hybrid	PV variable	Accuracy, Stability	Combines GA, ANFIS, Sliding Mode, or IncCond
[21–27]	FLC / Fuzzy-PID / Type-2 / Hybrid	Nonlinear or Active Loads	THD, Power Quality, Dynamic Response	Enhanced performance, robustness; still limited load evaluation in some
This Work	FLC + Firefly Algorithm	Resistive, Inductive, Nonlinear	MSE, THD, Dynamic Response	Simultaneous optimization of input/output MFs; practical improvement over previous works

Even though a number of research studies have been carried out with significant advances in optimizing fuzzy controllers using GA, PSO, and FA, most of the previous works have limited system performance evaluation to specific load conditions or purely resistive loads. For this work, the Firefly Algorithm will be employed for optimizing the input and output membership functions of the three-phase inverter fuzzy controller in order to improve system performance for variable load conditions, including resistive, inductive, and nonlinear loads. The approach simultaneously optimizes MSE, THD, and dynamic response, hence making it practical and different from the earlier works.

## Fuzzy logic controller design

Different control methods for the inverters in the photovoltaic systems have been presented to obtain an AC voltage from the generated DC voltage. Each of these methods has certain merits and demerits. In this paper, a fuzzy logic-based control is applied. The most important and critical part for this kind of approach lies in the design of the fuzzy controller’s membership functions. Poorly designed membership functions lead to deterioration in system performance or even instability. For that reason, optimization of these membership functions should benefit from expert knowledge and intelligent algorithms.

Fuzzy logic was first introduced in the 1960s by Dr. Lotfi Zadeh, a Professor in the Computer Science Department of the University of California, Berkeley. His classic work on fuzzy sets, published in 1965, opened an entirely new direction in systems engineering and computer science. Fuzzy logic explicitly expresses the fact that most of the problems that have been related to scientific and engineering areas are ambiguous. While the conventional approach tries to reduce uncertainty to aim for higher accuracy and efficiency, Zadeh proposed the development of models, which explicitly introduce ambiguity as an integral part of the system itself.

Usually when designing and implementing a Fuzzy Logic Controller (FLC) designers depend significantly on personal knowledge and experience or that of able engineers/environmental science specialists. This helps establish a working draft or initial Fuzzy Logic Controller (FLC) concept. The next phase utilizes control engineering concepts. Similar to designing conventional controllers, one can develop the design for an FLC. Both rely on results from the choice of parameters and subsequent controller performance. Currently there is work being done on establishing similarities between FLCs and PID controllers and also how to enhance these devices.

Fuzzy controllers use “fuzzy logic” (based on heuristics) rather than traditional mathematical techniques to achieve desired results through a variety of techniques including fuzzification, membership functions, fuzzy rules, and programmed inference mechanisms. In FLCs, input variables include error and change in error. These are used as inputs to the fuzzy controller in the context of “closed loop control”, in which a feedback mechanism continually compares output to reference input. The four main parts to a fuzzy logic controller are as follows^28^:

- Fuzzification.
- Fuzzy Rule Base,
- Inference Mechanism,
- Defuzzification.

**Fig. 1:**
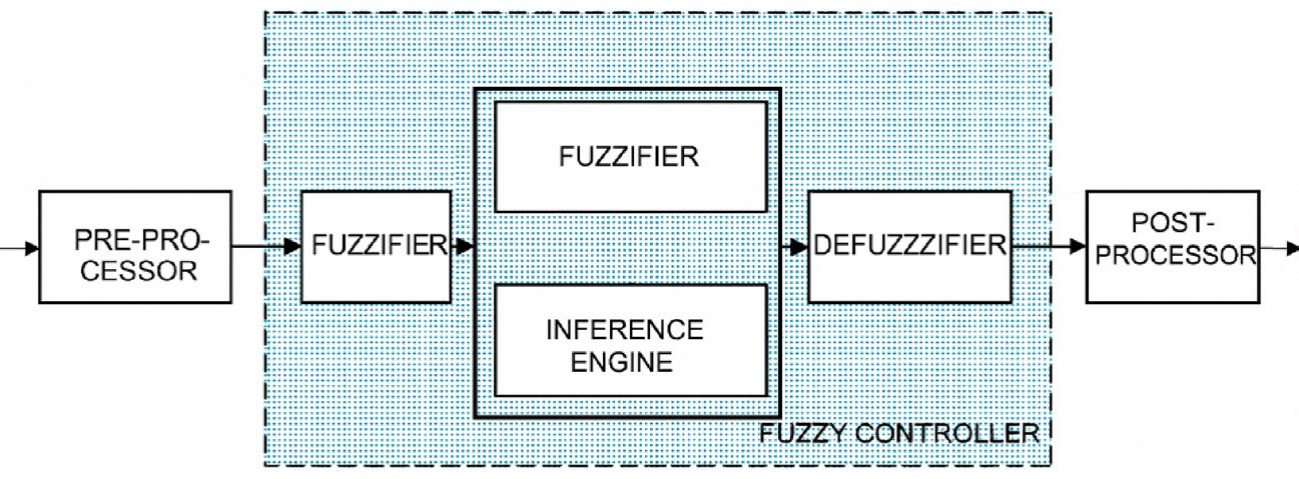
Fuzzy Controller^28^.

Continuous signals are converted into fuzzy whole numbers and back again through fuzzification and defuzzification. The inference process provides the mapping from the fuzzy input variables to the corresponding output control actions. The structure of a typical fuzzy controller is shown in Fig. 1.

### Fuzzification

At this stage of the fuzzy system, it is necessary to establish what are the inputs and outputs that come from real life. To accurately create the if–then rules for the fuzzy system, the raw data were referenced to create the fuzzy membership functions. Once the membership functions have been established, the information can be used to apply fuzzy logic to the appropriate fuzzy system or network. A few examples of traditional fuzzifiers are listed below [29]:

- Singleton fuzzifier.
- Triangular fuzzifier.
- Gaussian fuzzifier.

### Fuzzy inference engine

To convert fuzzy set *A* in *U* into fuzzy set *B* in *V* using fuzzy inference subsystem, fuzzy logic maps set A into B using the relationship between the fuzzy sets represented by if–then rules defined within the Rule Base. Several different types of inference engines exist such as [29]:

- Mamdani inference engine.
- Lukasiewicz inference engine.
- Product inference engine.
- Min inference engine.

Once the inputs are fed into the inference engine, all the if–then rules will be processed to determine their degree of truth for the given input; if there is no complete match between the input and any of the rules, the inference engine will generate an appropriate output based on those rules which provided partial matches. Various methods exist to associate a partial match with an output.

### Rule base

An if-then fuzzy rule set comprises the main component of the fuzzing algorithm. This is the rule set that lays the foundation for the fuzzy system and provides a basis on which the other parts of the fuzzy system can operate and take action based on the results obtained through inference. The combination of these results from the inference engine will yield a final output, and because of differing consequences that all come from using each of the several ambiguous rules, all of the rules should be taken into consideration when making a decision.

### Defuzzification

This stage converts the fuzzy output obtained from the previous step into a usable crisp value. Converting a fuzzy set into a usable value in this step is rather complicated, as a fuzzy set cannot be directly interpreted as a usable value. Defuzzification represents a major step in the FIS because most controllers of physical systems require discrete signals. Some common methods of defuzzification are [30]:

- Maximum (Maxima) method.
- Center of average method.
- Center of gravity (Centroid) method.

## Firefly algorithm

Fireflies tend to group together in nature, with the less luminescent fireflies gravitating towards those with more luminescence. The Firefly Algorithm (FA) includes three fundamental assumptions concerning this behavior of fireflies [31]:

1. Female and male fireflies are similar in appearance; therefore, they do not distinguish between one another.
2. The more luminescent the firefly, the more attractive it is to other fireflies.
3. The brightness of a firefly serves as a measure of the Firefly Algorithm’s (static) objective function value in the optimization process.

For a maximization problem, the light intensity *I* of a firefly at position x is determined by $\:I\left(x\right)\propto\:f\left(x\right)$. The attractiveness *β* is a relative measure, because it has to be seen and perceived by other fireflies within a neighborhood. Therefore, the attractiveness is a function of the distance r_ij_ between firefly *i* and firefly *j*. Furthermore, the intensity of light decreases with distance from its source. As light is also absorbed by the medium, the attractiveness needs to be decreased by the absorption coefficient as well.

The above-defined distance *r* is not restricted to Euclidean distance, though other forms of distance measures can be defined in multidimensional spaces depending on the problem. In the case of a scheduling problem, for example, *r* can be treated as time delay or temporal distance. For complex networks, such as the Internet or social networks, *r* can be defined as a combination of local clustering degree and average proximity of nodes. Generally speaking, any feature that effectively represents the degree of attraction can be treated as the distance *r* in firefly algorithms.

1 $$\:I\left(r\right)=\frac{{I}_{s}}{{r}^{2}}$$

where *I*_*s*_ is the source light intensity. In a medium with a constant light absorption coefficient *γ*, the light intensity *I* varies with distance *r* according to:

2 $$I={I_0}{e^{ - \gamma r}}$$

The parameter *I*_*0*_ denotes the initial light intensity. In order to remove the singularity at *r* = 0 in expression $\:\frac{{I}_{s}}{{r}^{2}}$, one may approximate the combined effects of the inverse-square law and of the medium absorption by the following Gaussian form:

3 $$I(r)={I_0}{e^{ - \gamma {r^2}}}$$

Since the attractiveness of a firefly is proportional to the light intensity perceived by its neighboring fireflies, the attractiveness *β* can be defined as follows:

4 $$\beta ={\beta _0}{e^{ - \gamma {r^2}}}$$

where *β*_*0*_ indicates attractiveness at *r* = 0. In practical implementations, the attractiveness function *β(r)* can be represented by any monotonically decreasing function, such as the general form presented below:

5 $$B(r)={\beta _0}{e^{ - \gamma {r^m}}},\left( {{\mathrm{m}} \geqslant {\mathrm{1}}} \right)$$

A characteristic distance ${{\boldsymbol{\Gamma}}}=1/\sqrt \gamma$ can be defined such that the attractiveness decreases significantly from *β*_*0*_ to${\beta _0}{e^{ - 1}}$. For a constant absorption coefficient γ the characteristic length becomes:

6 $$\Gamma ={\gamma ^{ - 1/m}}~ \to {\text{1, m}}$$

The characteristic scale Γ should be related to the relevant scale in an optimization problem. If Γ is a fixed scale for a given optimization problem, and if the number of fireflies is large enough such that $n\gg m$ (where mmm is the number of local optima), the initial locations of these n fireflies should distribute relatively uniformly throughout the entire search space. During the iterations, fireflies are expected to move into the regions of all local optima. After comparing the best solutions among these local optima, the global optimum can be found efficiently. Recent studies show that the Firefly Algorithm can find the global optimum accurately when *n→∞* and the number of iterations $t\gg 1$. On the other hand, for a characteristic length *Γ* in an optimization problem, the parameter *γ* can be used as an appropriate initial value [31].

7 $$\gamma =\frac{1}{{{\Gamma ^m}}}$$

The distance between any two fireflies, *i* and *j*, located at positions $\:{x}_{i}$​ and $\:{x}_{j}$​ can be defined as a Cartesian distance:

8 $${r_{ij}}=~{x_i} - {x_j}=\sqrt {\mathop \sum \limits_{{k=1}}^{d} ({x_{i,k}} - {x_{j,k}})}$$

where, $\:{x}_{i,k}$​ denotes the *k-th* component of the position vector $\:{x}_{i}$ of firefly *i*. In two-dimensional space, this distance can be determined as:

9 $${r_{ij}}=~\sqrt {{{({x_i} - {x_j})}^2}+{{({y_i} - {y_j})}^2}}$$

The movement of a firefly *i* towards a more attractive firefly *j* is defined as follows:

10 $${x_i}=~{x_i}+{\beta _0}{e^{ - \gamma r_{{ij}}^{2}}}({x_j} - {x_i})+\alpha ~{\epsilon _i}$$

In Eq. (10), the second term describes the attractiveness and may be considered as the movement of firefly *i* towards the more attractive (brighter) firefly *j*, while the third term is the randomization component. where, $\:{\:\epsilon}_{i}$is a vector of random numbers drawn from either a Gaussian or a uniform distribution. For example, the simplest form of $\:{\:\epsilon}_{i}$can be written as rand - ½, where rand generates a uniformly distributed random number in the interval *[0*,*1]*. In most implementations, it is convenient to set *β*_*0*_ *= 1* and $\alpha \in [0,1]$. The parameter γ controls the type of attractiveness and influences the convergence rate and overall behavior of the Firefly Algorithm. Though theoretically γ can range from 0 to infinity, in practice, γ is usually around *O(1)*, which relates to the characteristic length *Γ*. Thus, for most applications, γ usually varies between 0.1 and 10.

There are two special cases when γ→∞ and when γ→0. In the case of γ→0, the attractiveness becomes constant, i.e., *β = β*_*0*_, and the characteristic length Γ→∞. This essentially means that the light intensity never drops to zero in an ideal environment; therefore, any flashing firefly can be seen anywhere within the domain. This implies that the global optimum can easily be reached if a flashing firefly occurs. Furthermore, if the inner loop over *j* is removed and *x*_*j*_ is replaced by the current global best g, the Firefly Algorithm becomes a special case of PSO. The performance of this special case is similar to the performance of the standard PSO.

In other words, the case of γ→∞ leads to Γ→0 and β(r)→δ(r), where *δ(r)* is the Dirac delta function. Thus, at the point that *Γ* approaches zero, that means that fireflies will have little or no attractiveness to each other. This forms the basis for that case where fireflies are wandering around in a foggy area with no ability to view one another; therefore, every firefly will be moving around haphazardly and totally at random. Thus, this definition represents a completely random means of searching. Consequently, at the end of this section if we use the previous mathematical relationship supply us with a means to increase the effectiveness of covered areas by altering the parameter *α* and reduce this by a gradual amount as it nears the optimal search locations. For instance, the following equation can be used to supply a similar methodology:

11 $$\alpha ={\alpha _\infty }+({\alpha _0} - {\alpha _\infty }){e^{ - t}}$$

where t∈[0,t_max_] is approximately the time of simulation, and t_max_ denotes the maximum number of generations. The variable α_0_ in the equation denotes the parameter of randomization at the beginning, while α_∞_ denotes the final value. An analogous function could, in principle, be applied in the situation concerning scheduling or feedback optimization:

12 $${{\boldsymbol{\upalpha}}}=~{{{\boldsymbol{\upalpha}}}_0}{{{\boldsymbol{\uptheta}}}^{\mathrm{t}}}$$

**Fig. 2:**
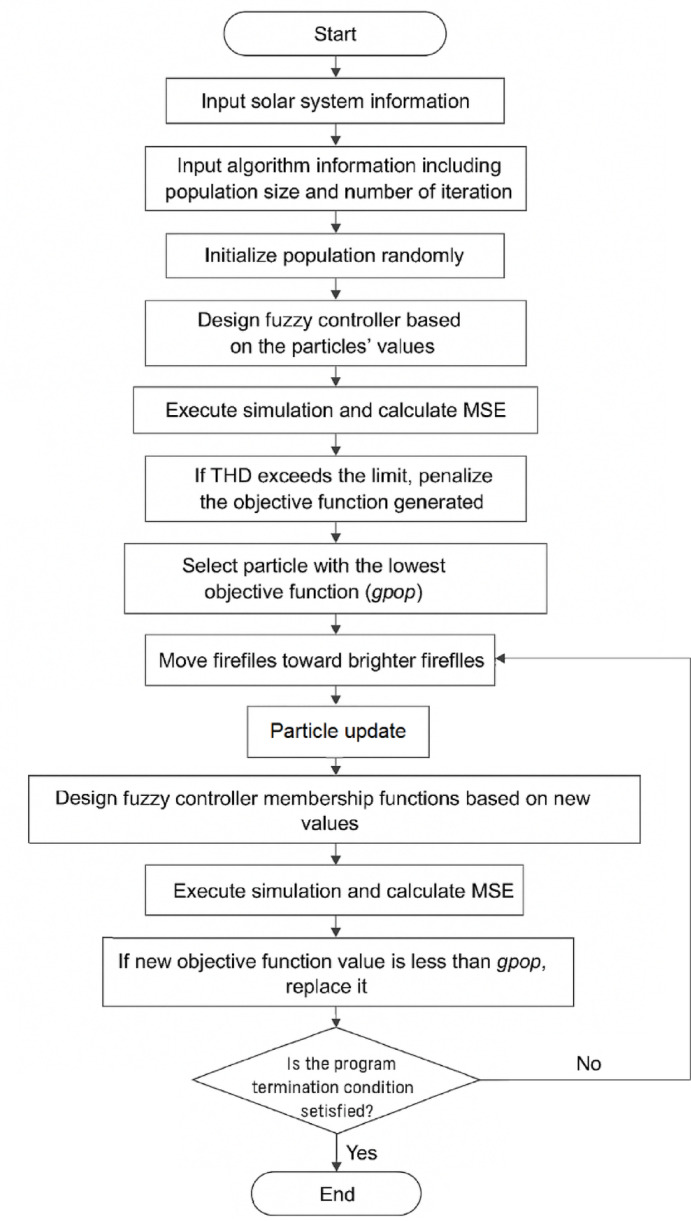
Flowchart of the proposed method.

Where θ∈(0,1] represents the constant affecting the speed of reduction of the randomization process. The process of the Firefly Algorithm in finding the optimal parameters of the new controller can be seen in the flowchart in Fig. 2.

The Firefly Algorithm (FA) is used to optimize the parameters in the design of the proposed fuzzy control system based on the Mean Squared Error (MSE) function. The procedure can be briefly described as follows:

### Step 1

Decide on the model of photovoltaic system to investigate. The elements required in such a model are the photovoltaic panel inverter, DC-DC converters, and so on.

### Step 2

To start with, there are parameters to be determined in terms of the firefly algorithms. These parameters include the number of fireflies and the upper limit.

### Step 3

It creates the group of fireflies, which occurs randomly in such a manner that all fireflies represent the solution and create the membership function.

### Step 4

For every firefly, calculations in function (MSE) are performed through simulations.

### Step 5

Determine the global-best or g_best_​ solution based on the value of the objective function.

### Step 6

Next, the locations of fireflies are moved based upon attractiveness and the global optimal solution obtained so far.

### Step 7

If the end conditions are not satisfied, return to Step 4.

### Step 8

End the algorithm.

In other words, this process further optimizes the membership functions so that there is optimal performance in terms of voltage error, dynamic performance, and total harmonic distortion.

In the optimization process, the firefly algorithm considers the parameters of the fuzzy controller’s membership functions as control variables. More specifically, these control variables are the positions, widths, and shapes of all input and output membership functions. In this way, the firefly algorithm seeks to minimize the mean squared error of the output voltage, decrease the total harmonic distortion, and improve the dynamics of the inverter under various load conditions such as resistive, inductive, and nonlinear loading.

## Simulation and results analysis

**Fig. 3:**
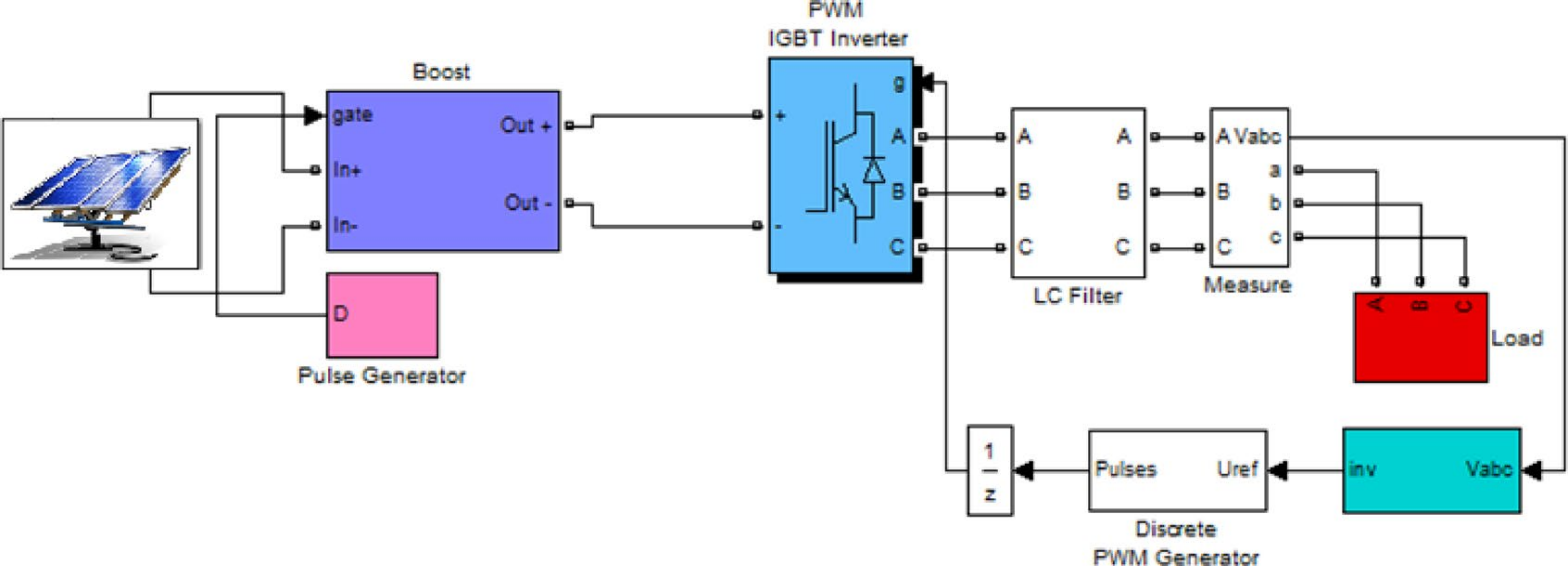
Studied photovoltaic system.

**Fig. 4:**
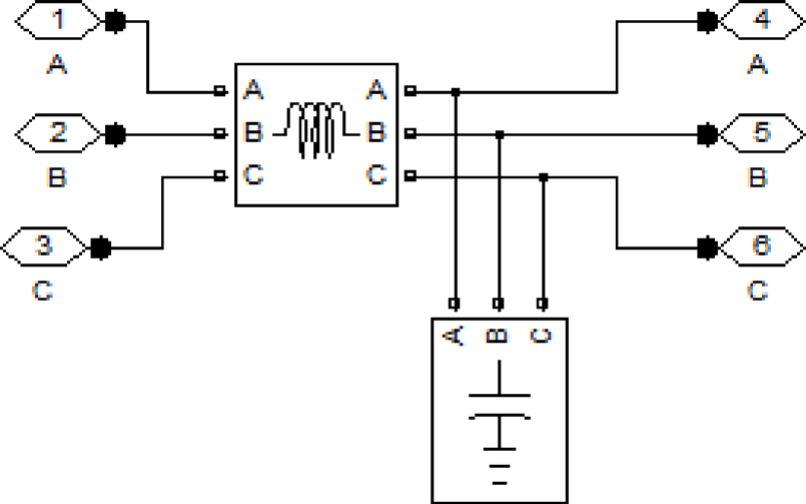
LC filter circuit model.

This section presents the results obtained from simulations performed in the MATLAB environment. For the simulations, the photovoltaic system shown in Fig. (3) is selected as the test system. The system consists of a solar array whose output voltage is boosted using a DC–DC boost converter, and the boosted voltage is then converted to AC using a three-phase inverter. A three-phase output filter is employed at the inverter side to reduce harmonic components, as illustrated in the circuit model of the designed filter in Fig. (4). Finally, the regulated AC power is supplied to three-phase loads.

**Fig. 5:**
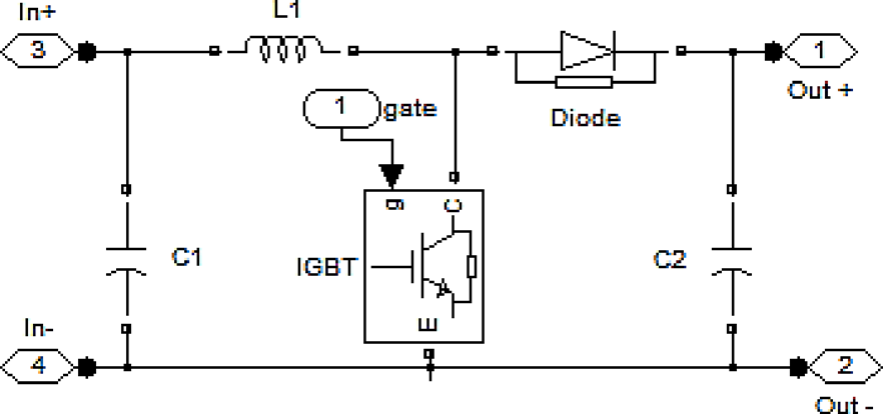
Boost converter circuit model.

The equivalent circuit of the designed boost converter is shown in Fig. 5.

The parameters of the three-phase PWM inverter in the simulation are shown in Table 2.

**Table 2:** Inverter parameters.

Parameter	Symbol	Value
Switching period	Ts​ (s)	2 × 10⁻⁶
Filter time constant	Tf​ (s)	1 × 10⁻⁶
DC link voltage	Vd​ (V)	0
AC Voltage	Vf​ (V)	0
Capacitance	Cs​ (F)	0.001
On-State Resistance	Ron​ (Ω)	∞
Series Resistance	Rs​ (Ω)	5000

**Fig. 6:**
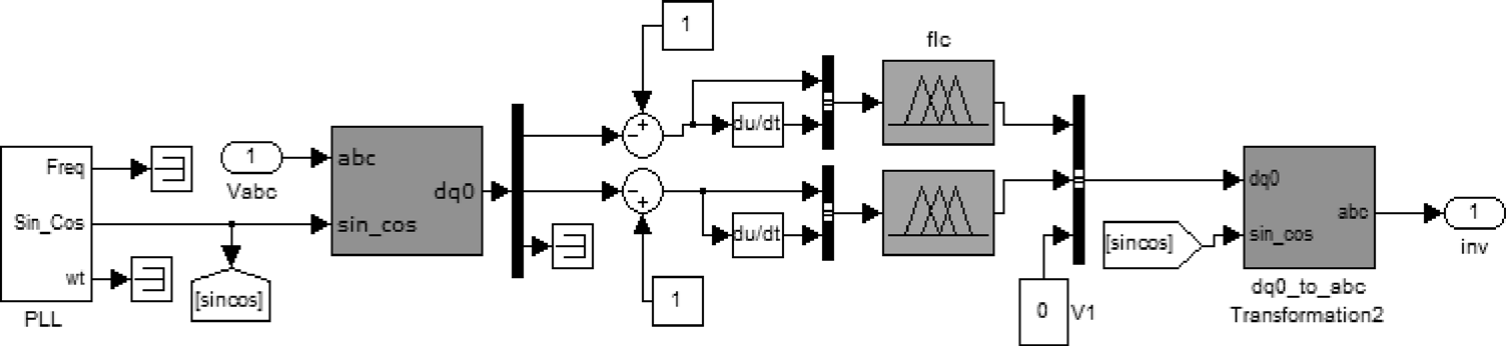
Fuzzy inverter control circuit.

The inverter controller uses fuzzy logic. Figure 6 illustrates the circuit model of the fuzzy controller.

**Fig. 7:**
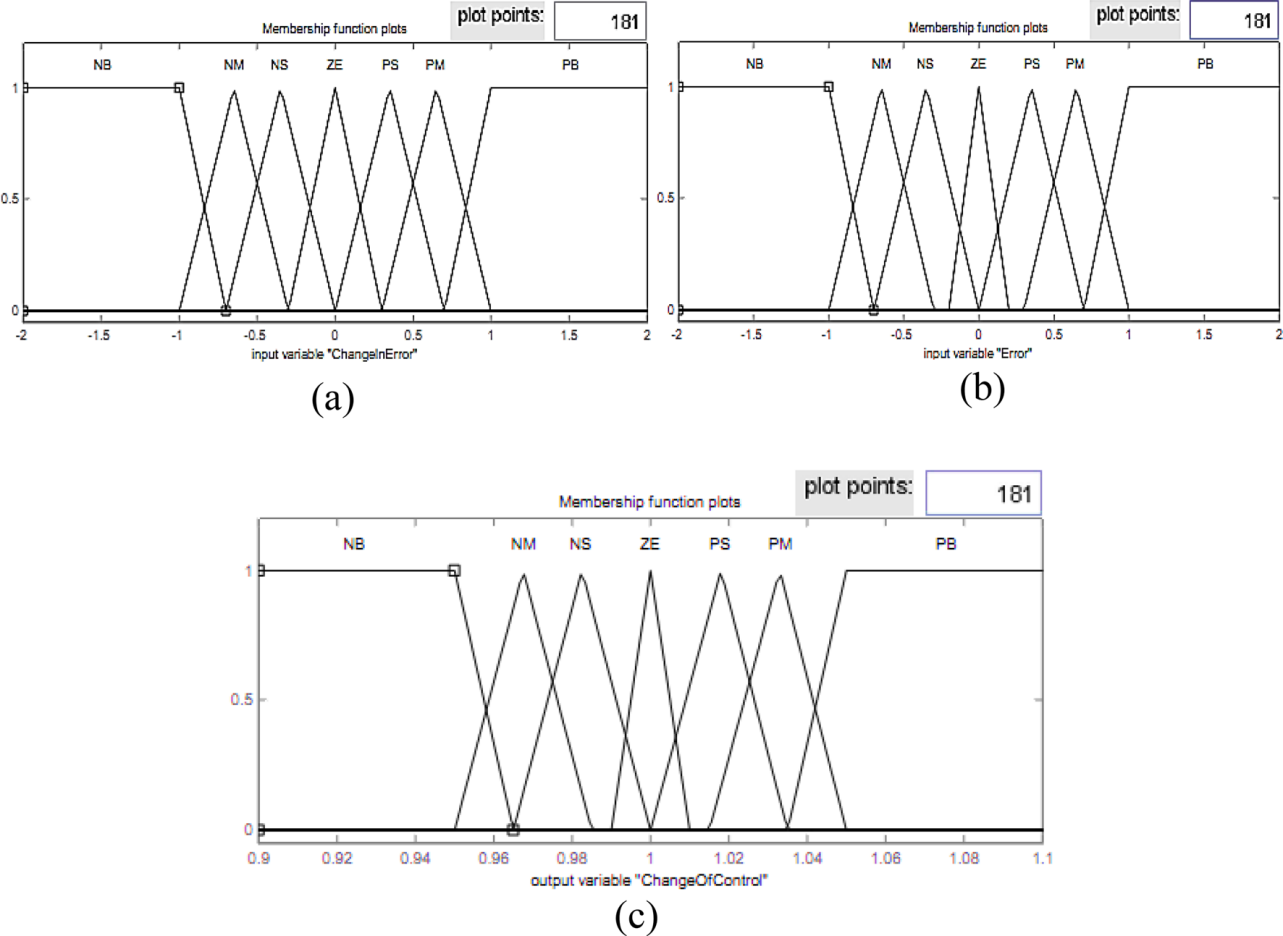
Input and output membership functions of the fuzzy controller for V_d_.

**Fig. 8:**
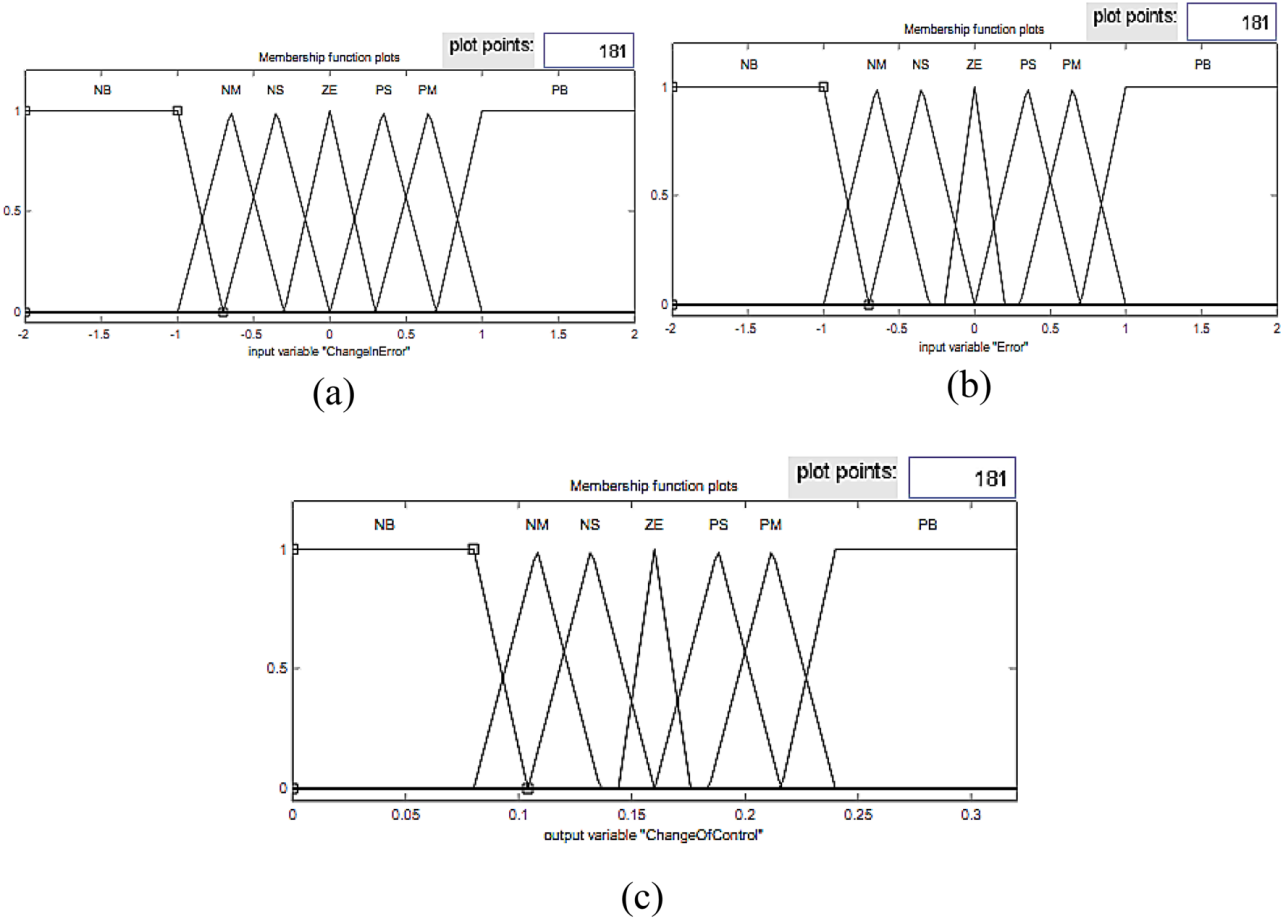
Input and output membership functions of the fuzzy controller for V_q_.

The sampled voltage is transformed into the *dqo* reference frame using the Park transformation. The inputs of the fuzzy controller are the error and the derivative of the error; therefore, the outputs of these are transmitted to the pulse generator circuit in order to produce the corresponding voltages. Note that the *d* and *q* fuzzy controller input and output membership functions are presented in Figs. 7 and 8, respectively.

The design of the fuzzy logic controller is based on careful selection of input and output variables, specifically the voltage error and its derivative as inputs, and the corresponding control signals as outputs. The shape, number, and range of membership functions significantly influence the controller’s ability to capture the nonlinear dynamics of the inverter system. Triangular and Gaussian membership functions were used to provide smooth control transitions, while the number of linguistic levels was chosen to balance computational complexity and control resolution. Optimizing these membership functions using the Firefly Algorithm ensures that the controller can accurately track the reference voltage, minimize voltage fluctuations, and maintain low total harmonic distortion (THD) across varying load conditions. Poorly designed membership functions would lead to slower response, higher overshoot, and increased harmonic content, highlighting the critical impact of proper membership function tuning on overall system performance.

In this study, Firefly Algorithm (FA), Particle Swarm Optimization (PSO), and Genetic Algorithm (GA) are employed to optimize the membership function designs of the controllers. The parameters of these algorithms are shown in Table 3.

**Table 3:** Algorithm parameters.

Algorithm	Parameter	Value
**Genetic Algorithm (GA)**	Mutation Rate	0.03
	Mutation	0.3
	Crossover	0.8
	Iterations	30
	Population	100
**Particle Swarm Optimization (PSO)**	W	0.7
	Vmin	0.9
	Vmax	0.4
	C1 = C2	2
	Iterations	30
	Population	100
**Firefly Algorithm (FA)**	ρ (Light Absorption)	0.65
	α (Randomization)	0.85
	β (Attractiveness)	0.7
	Iterations	30
	Population	100

The parameters of GA, PSO, and FA algorithms in Table 3 are based on the common values of the parameters reported in the literature. The parameters of the GA algorithm are based on the values reported in [7,13], the parameters of the PSO algorithm are based on the values reported in [28,29], and the parameters of the FA algorithm are based on the values reported in [31].

In the process of optimization, the Mean Squared Error (MSE) measure is used, as shown in Eq. (13).

13 $$MSE=\frac{{\sum\limits_{{i=1}}^{l} {{{({V_{dref}} - {V_d})}^2}+{{({V_{qref}} - {V_q})}^2}} }}{l}$$

**Fig. 9:**
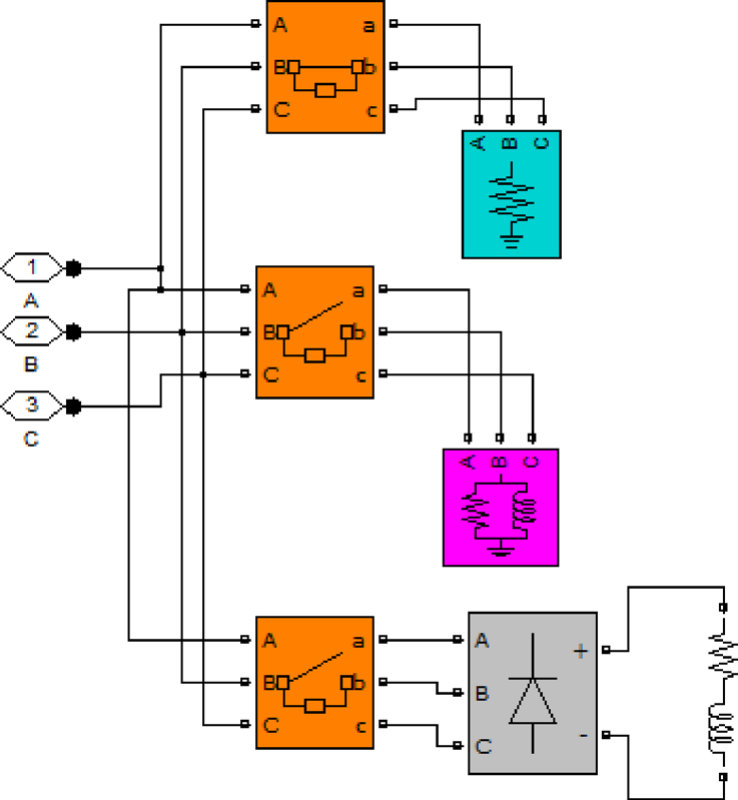
Simulated load model.

In the equation, *l* represents the number of sample points. The experiments were performed under different loading conditions. To begin with, the 50 kW resistive load was applied to the system. At time *t = 0.1* s, the 50 kW load was turned off, and the RL load was connected to the inverter with active power of 50 kW and inductive reactive power of 5 kVAR. Later, at *t = 0.2* s, the load was turned off, and the nonlinear load was applied. The proposed load model is shown in Fig. 9. Three different controllers—conventional PI controller, non-optimized fuzzy controller, and optimized fuzzy controller—were applied separately to the proposed controller and compared. For the conventional PI controller, the parameters *K*_*p*_*​=0.4* and *K*_*i*_*​=1.7* were applied.

**Fig. 10:**
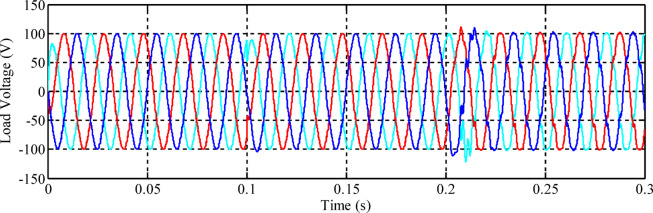
Load voltage using the GA-optimized fuzzy controller.

To evaluate the performance of the control systems, the three-phase voltage waveforms for each designed controller are presented. Figure 10 illustrates the inverter output voltage when the fuzzy controller, optimized using the Genetic Algorithm (GA), is employed.

**Fig. 11:**
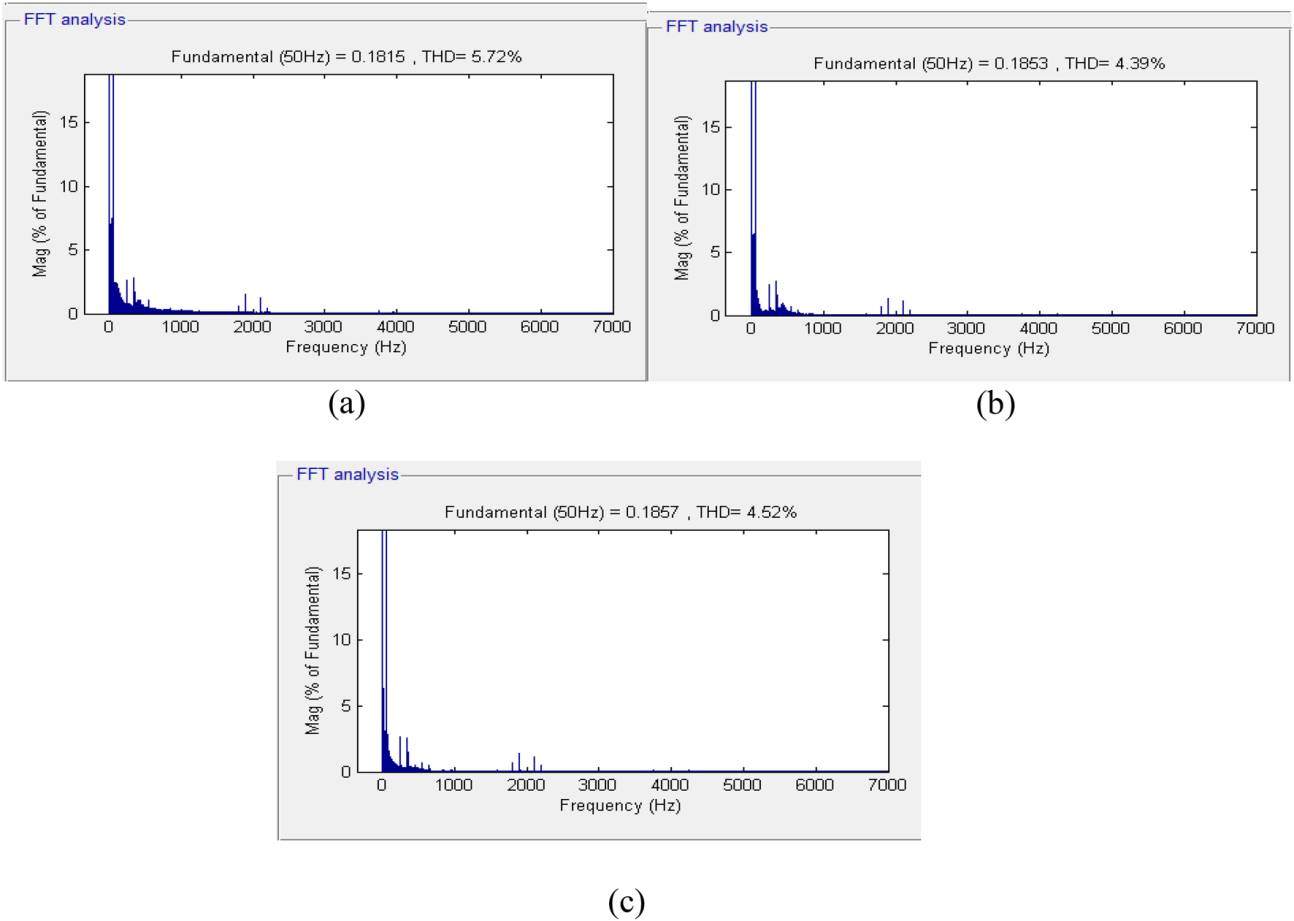
Fourier expansion of voltage signals for GA-optimized fuzzy controller: (a) Phase A, (b) Phase B, and (c) Phase C.

Under different loading conditions, the voltages in all three phases vary, especially when the nonlinear load is applied to the power system at time *t = 0.2 s*. To ensure the quality of the inverter output waveforms, Fast Fourier Transform analysis was performed, and the value of Total Harmonic Distortion was obtained. The quality of the inverter output waveform varies inversely with the percentage value of the THD. To meet standards specified by IEEE 929–2000, the value of the output waveform distortion should not exceed 5% in all cases. The THD in the voltages of all three phases using the GA-optimized fuzzy controller is presented in Fig. 11.

**Fig. 12:**
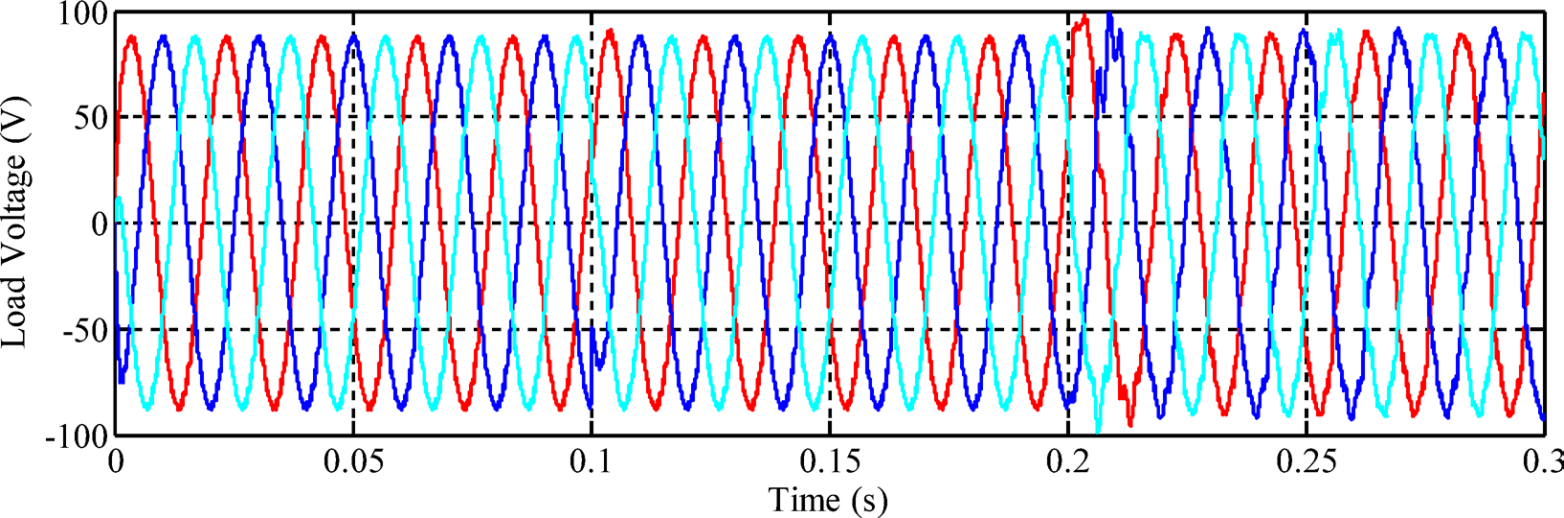
Load voltage using the non-optimized fuzzy controller.

The total harmonic distortion (THD) in the voltages was determined after the 15th cycle. The data obtained reveal that the THD in the phase B voltage is 5.72%, which exceeds the specified THD limitation in distribution networks. The THD in Phases A and C voltages stands at 4.39% and 4.52%, respectively, which are somewhat high. Later, the fuzzy inverter was developed employing Particle Swarm Optimization (PSO) and connected to the inverter controller circuit. Three-phase voltages at the load end are shown in Fig. 12.

**Fig. 13:**
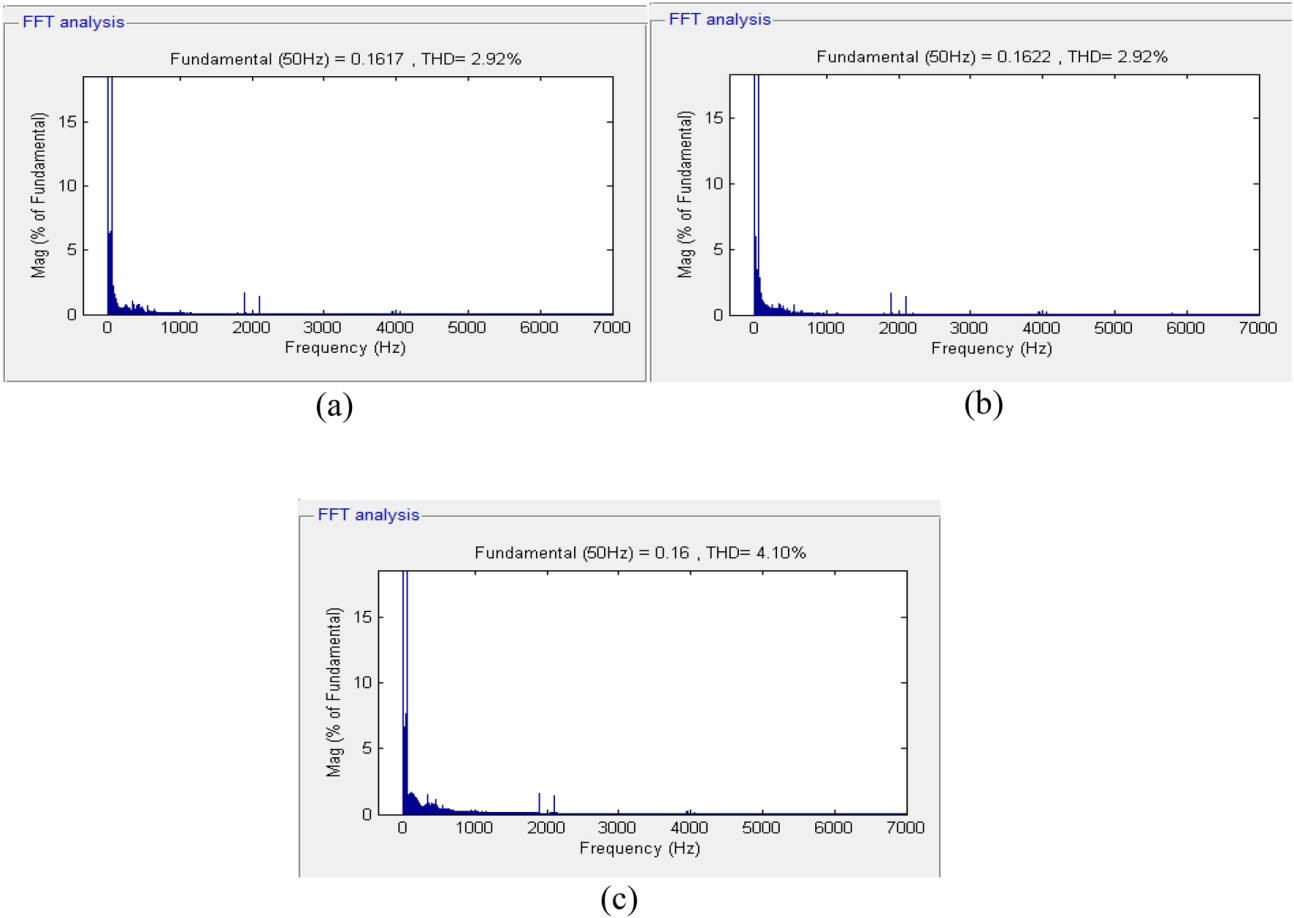
Fourier expansion of the voltage signals for non-optimized fuzzy controller: (a) Phase A, (b) Phase B, and (c) Phase C.

At the moment when the load changes from the resistive to the *R*_*L*_ load at *t = 0.1* s, there are small fluctuations in the phase voltages, but they rapidly stabilize at their nominal values. At *t = 0.2* s, due to the connection of the nonlinear load, there are oscillations in the phase voltages, but these persist for less than a quarter of a cycle and rapidly decay. The amplitude level of the voltages remains the same, and the inverter output frequency is retained at 50 Hz. Later, the total harmonic distortion (THD) of the voltages applied to the loads, using the non-optimized fuzzy controller, is depicted in Fig. 13. It needs to be mentioned that the calculation of the THD is performed after 15 cycles.

**Fig. 14:**
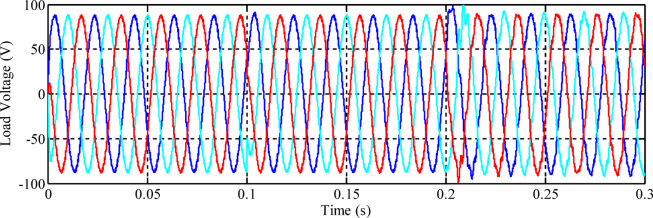
Load voltage using the FA-optimized fuzzy controller.

**Fig. 15:**
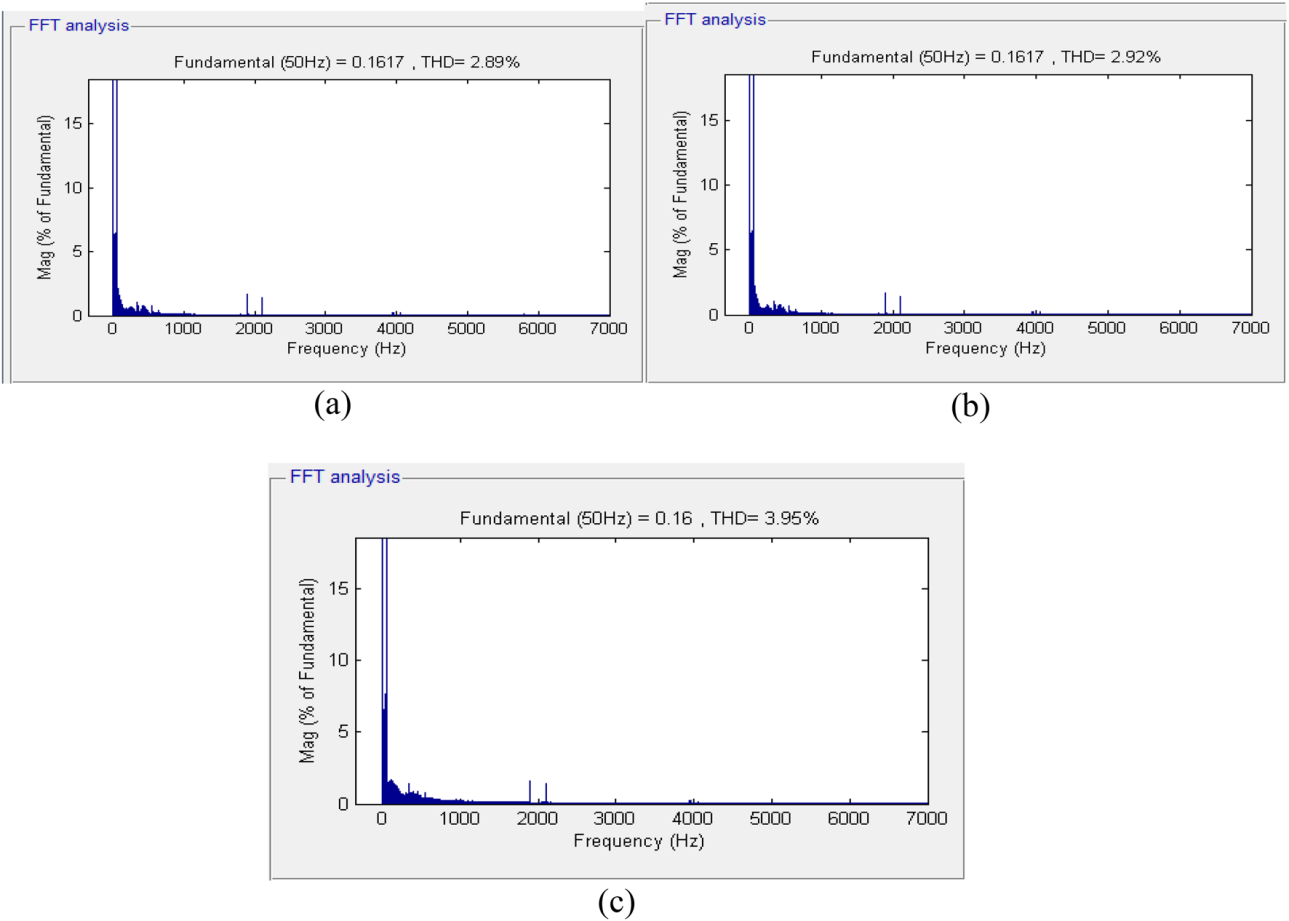
Fourier expansion of the voltage signals for FA-optimized fuzzy controller: (a) Phase A, (b) Phase B, and (c) Phase C.

Figure 14 above illustrates that the output voltage of the inverter is insensitive to changes in the load current. It can be observed that despite changes in the load current, there are transient changes in the output voltage, but its amplitude and frequency are constant with a phase shift of 120° between the phases. The controller has good reference voltage tracking characteristics and maintains proper performance criteria. The total harmonic distortion (THD) in the output voltage at the load bus using the optimized fuzzy controller developed using the Firefly Algorithm is shown in Fig. 15. Similar to the previous two controllers, the THD calculation considers 15 cycles.

Figure 14 illustrates that the output voltage of the inverter is insensitive to changes in the load current. It can be observed that despite changes in the load current, there are transient changes in the output voltage, but its amplitude and frequency are constant with a phase shift of 120° between the phases. The controller has good reference voltage tracking characteristics and maintains proper performance criteria. The proposed FA-based fuzzy logic controller has ensured an outstanding performance under non-linear loads and disturbances. When abrupt changes are caused on the system, from a resistive load to an RL load and then to a non-linear one, the controller keeps the three-phase output voltages at their nominal amplitude and frequency with a constant 120° phase shift. Similarly, the transients developed in the system due to the sudden variation in the applied loads are settled rapidly, i.e., in less than one-fourth of the cycle. Also, the Fast Fourier Transform analysis of the output voltages verifies the level of THD, which is kept at a definite and optimized value, i.e., 2.92%, 2.89%, and 3.95% for phases A, B, and C, respectively, in direct compliance with the IEEE 929–2000 standards, as these values are less than or equal to 5%. Also, the MSE of the optimal controller is significantly reduced to 0.0071, as compared to the results obtained by using non-optimized fuzzy techniques, i.e., to 0.0075, and to the results obtained by using non-optimized traditional PI control techniques, i.e., to 0.0112, affirming their high level of accuracy in reference voltage tracking.

The total harmonic distortion (THD) in the output voltage at the load bus using the optimized fuzzy controller developed using the Firefly Algorithm is shown in Fig. 15. Similar to the previous two controllers, the THD calculation considers 15 cycles.

Besides the results of voltage regulation, MSE, and THD, the convergence of the optimization algorithms has also been assessed. Table 4 shows the number of iterations taken by the Firefly Algorithm (FA), Particle Swarm Optimization (PSO), and Genetic Algorithm (GA) to converge to the optimal fuzzy controller design parameters. From the results, it is clear that the FA converges faster to the optimal solution while still producing a high-quality solution.

**Table 4:** Convergence comparison of FA, PSO, and GA.

Method	Iterations to Convergence	Final MSE	Final THD (%)
FA	25	0.0012	2.5
PSO	40	0.0013	2.7
GA	60	0.0014	2.8

## Results and discussion

**Fig. 16:**
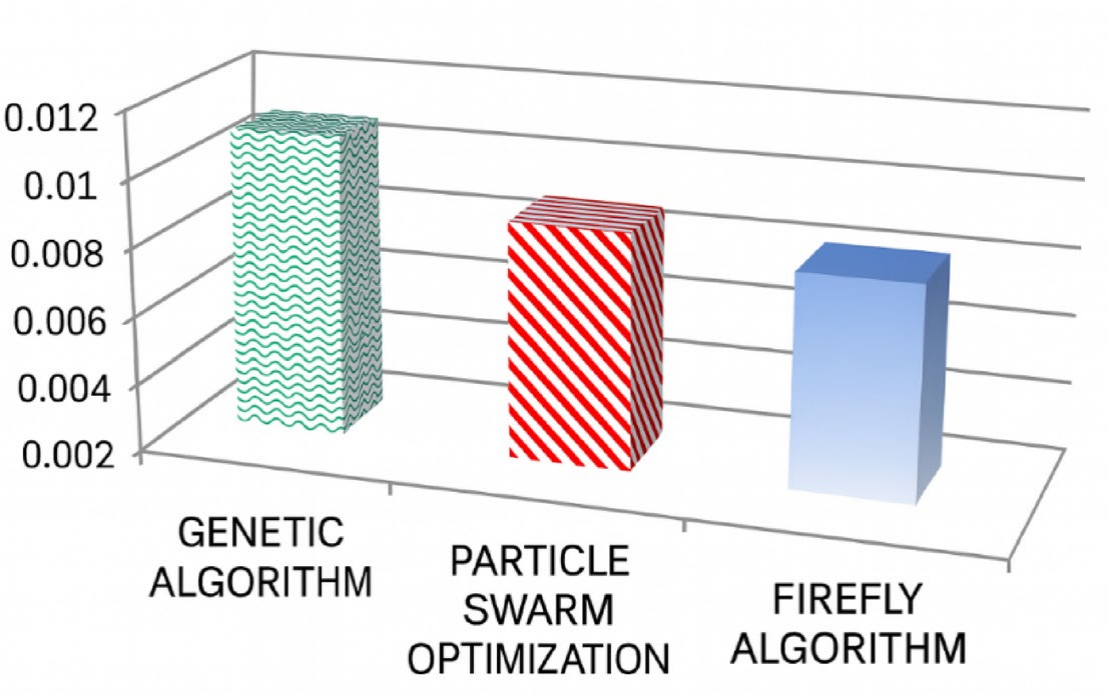
Mean Squared Error (MSE) values of different control methods.

This section presents a detailed simulation analysis of the proposed FA-optimized fuzzy logic controller, evaluating its performance under different load conditions and its impact on voltage regulation, dynamic response, and total harmonic distortion (THD). The total harmonic distortion at the load section is 2.92%, 2.89%, and 3.95% in phase A, phase B, and phase C, in that order. Next, the comparison analysis of the performance of these algorithms based on the result of the simulation analysis can proceed. For clarity, the mean squared error of these algorithms shall first be determined through the following calculations and represented in the bar chart in Fig. 16.

From the obtained result, the optimized fuzzy controller gives the smallest MSE value of 0.0071, whereas the inverter using the conventional PI controller gives an MSE value of 0.0112. In addition, the non-optimized fuzzy controller gives the objective function value of 0.0075. It can be deduced from these results that the smallest MSE value indicates the best controller performance. Table 5 is used to represent the quantitative comparison of all three optimization techniques. In Table 5, it is observed that the Firefly Algorithm has the least value of MSE and optimizes the system within less iteration; i.e., FA converges faster compared to other optimization techniques. While optimizing the system by the FA method, the least average value of THD is obtained compared to other optimization techniques used for improving power quality in the system.

**Table 5:** Comparative performance of FA, GA, and PSO.

Algorithm	Best MSE	Iterations to Convergence	Average THD (%)	Convergence Behavior
GA	0.0112	24	4.87	Slow, oscillatory
PSO	0.0075	18	4.21	Moderate, may stagnate
FA	0.0071	12	3.25	Fast, stable

The firefly algorithm has inherent benefits that make it more desirable for optimization of fuzzy logic controller membership functions. The attraction of the candidate solutions towards better solutions in the search space, following the attractive feature of the firefly algorithm, has been found to offer a wider window of convergence. This feature has significantly improved the level of global search while minimizing the chances of convergence to local optima. Furthermore, the exploration-exploitation of the search space, following the factor of randomization and the factor of absorption of the firefly algorithm, has considerably improved efficiency in the optimization of output membership functions as well as input membership functions. These benefits have impacted a remarkable performance of the fuzzy controller designed using the firefly algorithm optimization. The MSE has reduced to desirable values, the voltage regulation is improved, percent THD is reduced to acceptable values, and the responses to varying nonlinear load have been improved. The benefits have impacted considerably on reducing the instability of the stand-alone PV system.

The robustness of the proposed FA-optimized fuzzy logic controller is clearly demonstrated. The simulation results for the resistive, RL, and non-linear loads are presented in Table 6. The table shows that the output voltage amplitude is maintained within a range of ± 1% of the reference voltage level. Moreover, the output voltage level is maintained even if the load is changed from resistive to RL or non-linear types. The voltage level is restored within less than a quarter cycle. This shows the damping ability of the proposed controller. The total harmonic distortion (THD) level of the three-phase voltages is low. The mean squared error (MSE) is very low. This shows that the proposed controller is capable of efficiently controlling the voltage level.

**Table 6:** Simulation results of fa-optimized fuzzy controller under different loads.

Load Type	Active Power (kW)	Reactive Power (kVAR)	Voltage Regulation (%)	Settling Time (s)	THD (%) Phase A	THD (%) Phase B	THD (%) Phase C	MSE
Resistive (R)	50	0	± 1	0.01	2.90	2.85	2.88	0.0071
Inductive (RL)	50	5	± 1	0.02	2.95	2.90	2.92	0.0072
Non-linear	50	5	± 1	0.025	2.92	2.89	3.95	0.0073

In order to assess system stability and reliability even further, more simulations were carried out with sudden changes in system loads and parameters. The system load changes suddenly from 50 kW to 70 kW. The simulation results show that the proposed controller provides a stable voltage regulation without any oscillations and quickly returns the output voltage to its reference value. Moreover, consistent performance under different operating conditions demonstrates high reliability. From a maintainability viewpoint, the modular structure of the fuzzy controller and the offline optimization using the Firefly Algorithm allow easy re-tuning of membership functions, reducing maintenance complexity and facilitating practical implementation.

The effects of the FA-optimized fuzzy logic controller on power quality are substantial. As indicated in Table 7, the FA-optimized fuzzy logic controller ensures that the total harmonic distortion (THD) is less than 5% for all phases and satisfies the IEEE 929–2000 standard. Compared with the conventional PI controller and the non-optimized fuzzy logic controller, the proposed FA-optimized fuzzy logic controller has better voltage regulation, faster dynamic response, and smaller MSE. This verifies that the proposed method not only improves the inverter’s performance but also provides high-quality AC power.

**Table 7:** Comparison of power quality metrics for different controllers.

Controller Type	MSE	Voltage Regulation (%)	Settling Time (s)	THD (%) Phase A	THD (%) Phase B	THD (%) Phase C
Conventional PI	0.0112	± 2	0.03	4.39	5.72	4.52
Non-Optimized Fuzzy	0.0075	± 1.5	0.02	2.95	2.90	2.92
FA-Optimized Fuzzy	0.0071	± 1	0.02	2.92	2.89	3.95

The proposed FA-optimized fuzzy logic controller is seen to have better performance in comparison with traditional PI control and unoptimized fuzzy logic control in terms of voltage regulation, response, and reduced THD. In traditional PI control, high sensitivity is observed for parameter variations, and in traditional unoptimized fuzzy logic control, tuning is performed in a manual way, which is not an easy task. However, in the case of the proposed controller, the intelligent optimization is performed, which is advantageous. Disadvantage in using the proposed controller is that in offline optimization, complexity is observed in computation, but in actual time, it is seen to have low complexity.

The results presented in Tables 5, 6 and 7 show that the FA-optimized fuzzy logic controller outperforms the conventional PI and non-optimized fuzzy controllers in terms of performance, efficiency, and power quality. The MSE value is minimized, convergence is achieved quickly, voltage is regulated properly, and THD is reduced.

In order to further stress the originality and efficiency of the proposed method, a comparative study with some of the existing works is provided in Table 8. The comparison is conducted based on system configuration, control method, optimization technique, type of loads taken into consideration, and performance metrics. From the table, it can be noted that most of the existing works have been conducted on limited types of loads or on optimizing the controller partially, whereas the proposed FA-optimized fuzzy logic controller performs better with resistive, inductive, and nonlinear loads.

**Table 8:** Comparison of the proposed FA-optimized fuzzy controller with existing works.

Reference	System Type	Control Method	Optimization Technique	Load Types Considered	Best THD (%)	Error / MSE	Key Limitation
^21^	PV inverter	FLC	None	Resistive	4.8	–	No optimization, limited load
^15^	PV system	FLC + SVPWM	Golden Eagle Optimization	Resistive	3.9	–	No nonlinear load
^19^	Grid-connected PV	FLC	None	R, RL	4.2	–	No intelligent optimization
^24^	Active power filter	Fuzzy-PID	IFGO	Nonlinear	3.5	0.009	Not applied to inverter
^16^	PV inverter	FLC	PSO	Resistive	3.2	0.008	Single load type
**This work**	Standalone PV inverter	FLC	**Firefly Algorithm (FA)**	**R**,** RL**,** Nonlinear**	**2.89**	**0.0071**	–

## Conclusion

The presented work offers an optimized fuzzy controller design for a three-phase inverter in standalone photovoltaic (PV) power systems using the Firefly Algorithm (FA). The main objective of designing the controller is to optimize the input/output membership functions in order to reduce the mean squared error (MSE) of the output voltage, maintain stable voltage amplitude and frequency, and reduce the total harmonic distortion (THD) due to variable loads. Simulation outputs reveal that the FA-optimized fuzzy controller demonstrates stable performance and fast dynamic reaction with respect to resistive, inductive, and non-linear loading patterns. When compared with conventional proportional-integral (PI) controller designs and fuzzy controller designs optimized using Genetic Algorithm (GA) and Particle Swarm Optimization (PSO) algorithms, the FA methodology gives the smallest MSE value, maintains constant output voltage amplitude and frequency under varying loads, and quickly damped the transient level of load changes. In addition, the output voltages in all three-phases maintain less than 5% standard THD specifications and demonstrate significant improvements compared to other designs in order to support high power quality. Therefore, applying the Firefly Algorithm in the optimization of membership functions in the fuzzy controller makes less use of advanced mathematical modeling while improving tracking performance of the reference voltages and system stability. The presented approach presents an efficient and optimal solution in real photovoltaic power applications related to the control of three-phase inverters in standalone photovoltaic power systems. The obtained data from the presented work indicates the reliability and applicability of the presented design in real-world photovoltaic power applications. The simulation results validate that the proposed FA-optimized fuzzy logic controller enhances the performance of the standalone PV inverter, achieving a minimum THD of 2.89% and an MSE of 0.0071, thus ensuring better power quality and control accuracy than the existing methods.

In addition, future research will mainly focus on the real-time implementation of the developed FA-optimized fuzzy logic controller on various real-world hardware platforms like DSP and FPGA-based control systems. The robustness of the system will be tested by conducting experimental analysis on real-world environmental conditions like changes in solar irradiance, temperature changes, and uncertain loads. Moreover, the effect of sensor noise, delays, and inverter switching will also be considered. In addition, to enhance the real-world implementation of the presented control strategy, some online optimization methods can be considered for adjusting the membership functions based on changes in system parameters and aging.

## Data Availability

All data generated or analysed during this study are included in this published article.

## Abbreviation

PV: Photovoltaic
FLC: Fuzzy Logic Controller
GA: Genetic Algorithm
PSO: Particle Swarm Optimization
FA: Firefly Algorithm
THD: Total Harmonic Distortion
MSE: Mean Square Error
VSI: Voltage Source Inverter
PWM: Pulse Width Modulation
SVPWM: Space Vector Pulse Width Modulation
V_dc_​: DC-link voltage
V_abc_​: Three-phase output voltage
I_abc_​: Three-phase output current
e(t): Error signal
Δe(t): Change in error
P: Output power
f: Fundamental frequency
R: Resistive load
RL: Resistive–Inductive load
µ: Membership function
J: Objective (cost) function
n: Number of fireflies
β: Attractiveness coefficient
γ: Light absorption coefficient
α: Randomization parameter
